# Responses in left inferior frontal gyrus are altered for speech‐in‐noise processing, but not for clear speech in autism

**DOI:** 10.1002/brb3.2848

**Published:** 2022-12-27

**Authors:** Stefanie Schelinski, Katharina von Kriegstein

**Affiliations:** ^1^ Faculty of Psychology, Chair of Cognitive and Clinical Neuroscience Technische Universität Dresden Dresden Germany; ^2^ Max Planck Research Group Neural Mechanisms of Human Communication Max Planck Institute for Human Cognitive and Brain Sciences Leipzig Germany

**Keywords:** auditory, autism spectrum disorder, inferior frontal gyrus, speaker‐in‐noise recognition, speech‐in‐noise, speech recognition, voice identity recognition

## Abstract

**Introduction:**

Autistic individuals often have difficulties with recognizing what another person is saying in noisy conditions such as in a crowded classroom or a restaurant. The underlying neural mechanisms of this speech perception difficulty are unclear. In typically developed individuals, three cerebral cortex regions are particularly related to speech‐in‐noise perception: the left inferior frontal gyrus (IFG), the right insula, and the left inferior parietal lobule (IPL). Here, we tested whether responses in these cerebral cortex regions are altered in speech‐in‐noise perception in autism.

**Methods:**

Seventeen autistic adults and 17 typically developed controls (matched pairwise on age, sex, and IQ) performed an auditory‐only speech recognition task during functional magnetic resonance imaging (fMRI). Speech was presented either with noise (noise condition) or without noise (no noise condition, i.e., clear speech).

**Results:**

In the left IFG, blood‐oxygenation‐level‐dependent (BOLD) responses were higher in the control compared to the autism group for recognizing speech‐in‐noise compared to clear speech. For this contrast, both groups had similar response magnitudes in the right insula and left IPL. Additionally, we replicated previous findings that BOLD responses in speech‐related and auditory brain regions (including bilateral superior temporal sulcus and Heschl's gyrus) for clear speech were similar in both groups and that voice identity recognition was impaired for clear and noisy speech in autism.

**Discussion:**

Our findings show that in autism, the processing of speech is particularly reduced under noisy conditions in the left IFG—a dysfunction that might be important in explaining restricted speech comprehension in noisy environments.

## Graphical abstract

Recognizing what another person is saying in a noisy environment (i.e., speech‐in‐noise recognition) is an everyday challenging task, especially for people with autism. Using functional magnetic resonance imaging, we show that the left inferior frontal gyrus among a set of meta‐analytically delineated areas is altered in autism for speech‐in‐noise recognition. Our finding is specific for speech‐in‐noise recognition since cerebral cortex response for clear speech was on a neurotypical level in the autism group.

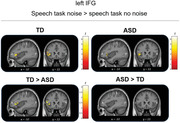

## 1 INTRODUCTION

Recognizing speech in a noisy environment (speech‐in‐noise recognition), such as on a crowded street or in a busy canteen, is an everyday challenging task. Background noise significantly diminishes speech recognition success in normal hearing individuals (e.g., Ross et al., 2007; Sumby & Pollack, 1954) and can impede communication and cognitive performance (for reviews see Klatte et al., 2013; Picard & Bradley, 2001; Szalma & Hancock, 2011; van der Kruk et al., 2017).

Speech‐in‐noise perception is often altered or impaired in people with autism spectrum disorder (ASD), a condition characterized by difficulties in social communication (Alcantara et al., 2004; American Psychiatric Association, 2013; Foxe et al., 2015; Groen et al., 2009; Irwin et al., 2011; Schelinski & von Kriegstein, 2020; Smith & Bennetto, 2007). The objectively measured speech‐in‐noise perception difficulties are also featured in subjective reports of people with ASD (Schelinski et al., 2017; Schreiter, 2020). In contrast, speech recognition abilities under low noise or no noise conditions (i.e., clear speech) are intact in ASD (Schelinski et al., 2016, 2014).

Only a few studies investigated the neural processing of speech‐in‐noise perception in ASD (Hernandez et al., 2020; Lin et al., 2021; Russo et al., 2009; Schelinski et al., 2022). These studies focused on alteration and reduction of responses in subcortical sensory structures (Russo et al., 2009; Schelinski et al., 2022) and/or did not include a speech recognition task (Hernandez et al., 2020; Lin et al., 2021; Russo et al., 2009). Russo et al. (2009) found altered brainstem responses in children with ASD as compared to typically developing children when passively listening to speech (i.e., syllables). These alterations were present both for speech without noise as well as when speech was presented with additional white noise. Schelinski et al. (2022) showed enhanced responses within a typically developed control group, when performing a speech recognition task when the speech signal was presented with noise versus no noise in the left and right inferior colliculus (IC). The IC is a nucleus of the subcortical auditory pathway associated with processing of spectro‐temporal voice acoustic features (e.g., Baumann et al., 2011; Griffiths et al., 2001; for review see Pannese et al., 2015). In the ASD group, this was only the case in the left, but not the right IC (but there was no interaction between noise and group). Whether such response alterations for speech‐in‐noise recognition are confined to the subcortical sensory pathway or are present also in the cerebral cortex are to‐date unknown.

In a recent meta‐analysis, three cerebral cortex areas have been identified that are particularly involved in recognizing speech‐in‐noise in typically developed individuals: left inferior frontal gyrus (left IFG), right insula, and left inferior parietal lobule (left IPL) (Alain et al., 2018). Our first aim was to investigate the hypothesis that speech‐in‐noise perception in ASD is associated with reduced responses of one or several of these three regions.

Previous studies showed that on the behavioral level, speech‐in‐noise recognition abilities can be impaired in ASD (Schelinski & von Kriegstein, 2020) but that performance in tests on speech‐in‐noise recognition can be variable depending on the experimental design (Alcantara et al., 2004). Besides speech‐in‐noise recognition difficulties, there is evidence that voice identity recognition is impaired in ASD (Boucher et al., 1998; Klin, 1991; Schelinski et al., 2016, 2014, 2017). For example, we showed in previous studies that voice identity recognition is impaired in ASD (Schelinski et al., 2016, 2014) and that this contrasted intact speech recognition performance in conditions without noise (i.e., clear speech). A second aim of the present study was to replicate these previous behavioral findings on speech and voice identity recognition and to investigate on how far group differences in voice identity recognition performance are affected by noise.

Seventeen adults with ASD and 17 typically developed adults (matched pairwise on age, sex, IQ, and handedness), participated in a functional magnetic resonance imaging (fMRI) experiment on auditory speech‐in‐noise recognition (speech‐in‐noise recognition experiment). During the speech‐in‐noise recognition experiment, participants performed speech recognition tasks on speech that was either presented with noise (noise condition) or without noise (no noise condition). We hypothesized to find reduced brain responses for speech recognition in noisy conditions in contrast to the no noise condition in the ASD group as compared to typically developed controls (a noise x group interaction) in one or more of the three regions of interest (left IFG, right insula, left IPL). The design also permitted to test for replication of comparable cerebral responses for ASD and controls for clear speech (Schelinski et al., 2016; Tryfon et al., 2018). For the no noise condition (i.e., clear speech), we did expect to find comparable brain responses in speech‐related brain areas (Schelinski et al., 2016; Tryfon et al., 2018).

Speakers who were presented during the speech‐in‐noise recognition experiment were familiarized to the participants during a speaker familiarization before the fMRI. Thus, besides testing behavioral speech recognition abilities, the design also allowed to investigate behavioral performance in voice identity recognition. To formally test this, we additionally included a voice identity recognition experiment that includes the speakers presented during the speech‐in‐noise recognition experiment. In the voice identity recognition experiment, we tested voice identity recognition abilities when the voice signal was presented with and without additional noise (noise condition/no noise condition). Based on previous findings (Schelinski et al., 2016, 2014, 2017), we hypothesized that in the no noise condition the ASD group would show significantly lower performance as compared to the control group for voice identity but not for speech recognition. For the noise conditions, we expected that the group differences (i.e., lower performance for the ASD as compared to the control group) would be particularly pronounced for voice identity recognition. Based on previous findings, we also expected that the ASD group would perform worse as compared to the control group in speech‐in‐noise recognition (Schelinski & von Kriegstein, 2020). However, speech‐in‐noise recognition abilities have also been found to be variable in ASD depending on the experimental design (Alcantara et al., 2004; Groen et al., 2009), indicating that speech‐in‐noise recognition performance could also be comparable between the ASD and the control group.

Investigating the neural processing of speech‐in‐noise perception in ASD is important, because it contributes to a better understanding of basic mechanisms related to communication difficulties in ASD. Further, investigating speech‐in‐noise processing in a clinical population fosters our understanding of speech perception in challenging conditions not only in atypical, but also in typically developed individuals.

## 2 METHODS

### 2.1 Participants

Seventeen adults with ASD participated in the ASD group and 17 typically developed (TD) adults participated in the TD group. The groups in each experiment were matched pairwise. Each TD group participant was matched to one participant in the ASD group with respect to gender (male or female), chronological age (age difference within each participant pair ≤ 3 years), handedness (right or left as assessed by a standard questionnaire; Oldfield, 1971), and intelligence quotient (IQ; Table 1; full‐scale IQ difference within each participant pair was maximally one standard deviation [15 IQ points]). IQ was assessed using the German adapted version of the Wechsler Adult Intelligence Scale (WAIS‐III; Wechsler, 1997; German version by von Aster, 2006). All participants had an IQ within the normal range or above (IQ > 85), indicating that all participants were on a “high‐functioning” cognitive level. Additionally, groups showed comparable concentration performances (d2 test of attention; Brickenkamp, 2002) (Table 1).

**TABLE 1:** Descriptive data for the ASD and the control group and group comparisons

Characteristic	ASD group (*n* = 17)		Control group (*n* = 17)		Group comparison	
Gender	14 male, 3 female		14 male, 3 female			
Handedness^a^	15 right, 2 left		15 right, 2 left			
	M	SD	M	SD	*p*	*d (r)*
Age	30.53	10.15	31.35	10.03	.813	0.081 (0.04)
Range	20–54		21–54			
WAIS‐III^b^ scales						
Full‐scale IQ	110.65	11.68	114.18	12.55	.402	0.291 (0.14)
Verbal IQ	111.47	11.30	113.71	11.92	.579	0.193 (0.10)
Performance IQ	107.53	14.26	111.47	12.82	.403	0.291 (0.14)
Verbal working memory	110.12	13.81	112.88	13.11	.554	0.205 (0,10)
d2 Test of attention^c^	104.24	14.07	107.12	7.17	.457	0.258 (0.13)
AQ^d^	37.41	8.65	16.12	5.31	< .001*	2.966 (0.83)

*Note*: Each participant in the control group was matched with respect to chronological age, gender, intelligence quotient (IQ), and handedness to the profile of one ASD group participant. Scores are summarized as average over group with standard deviation (SD) and *p*‐values and effect sizes (Cohen's *d*) from independent *t*‐tests.

Abbreviations: M ,  mean; SD,  standard deviation.

^a^ Handedness was assessed using the Edinburgh handedness questionnaire (Oldfield, 1971).

^b^ WAIS‐III = Wechsler Adult Intelligence Scale, 3^rd^ version (Wechsler, 1997; German adapted version: von Aster et al., 2006; M = 100; SD = 15).

^c^ d2 Test of attention (Brickenkamp, 2002; M = 100; SD = 10).

^d^ AQ, Autism Spectrum Quotient (Baron‐Cohen et al., 2001; German version adapted from Freitag et al., 2007). A total score of 32+ is considered a useful cut‐off for distinguishing individuals who have clinically relevant levels of traits associated with autism spectrum (Baron‐Cohen et al., 2001).

* Significant group difference (*p* < .05).

All participants were native German speakers. They reported normal hearing abilities and no limitations or disorders associated with the ear or hearing. Normal hearing abilities were confirmed with pure tone audiometry (hearing level equal or above 25 dB at the frequencies of 250, 500, 1000, 1500, 2000, 3000, 4000, 6000, and 8000 Hz tested in each ear separately).

All participants were free of psychotropic or any other medication at the time of testing. One TD group participant took a histamine antagonist for allergies when needed. One ASD group participant took antihypertensive medication and two ASD group participants took thyroid medication. None of the participants in the TD or ASD group reported to have a neurological disease.

We recruited people with ASD via autism outpatient clinics and announcements in communities for people with ASD, that is, self‐help groups and online fora. We attempted to maximize the number of clinical participants we could recruit in a given time (approximately 1 year). All ASD group participants had previously received a formal clinical diagnosis of Asperger syndrome (12 male, 3 female) or childhood autism (2 male, Verbal‐IQ 100 and 119) according to the diagnostic criteria of the International and Statistical Classification of Diseases and Related Health Problems (ICD‐10; World Health Organization, 2004). We only included participants into the ASD group who could provide a clinical diagnosis. That means that independent clinical experts made the diagnoses of all ASD participants before participating in the study. Additionally, the performance of all participants in the ASD group (except for one participant) was assessed with the Autism Diagnostic Observation Schedule (ADOS; Lord et al., 2000; German version by Rühl et al., 2004). ADOS data were not available for one participant. If caregivers were available (*n* = 11), we additionally conducted the Autism Diagnostic Interview‐Revised (ADI‐R; Lord, Rutter & Le Couteur, 1994; German version by Bölte et al., 2003) and the Social Communication Questionnaire (SCQ; Rutter et al., 2003; German version by Bölte & Poustka, 2006) (Table S1).

We recruited the TD group participants from the participant database of the Max Planck Institute for Human Cognitive and Brain Sciences, Leipzig, Germany. The database contains participants who have contacted the institute because they are interested in taking part in scientific studies. The database contains volunteers with, for example, different age ranges and different socioeconomic status or educational backgrounds. Participants in the TD group reported to have no neurological or psychiatric history and no family history of ASD. None of the TD group participants exhibited a clinically relevant number of traits associated with ASD as assessed by the autism spectrum quotient (AQ; Baron‐Cohen et al., 2001; German version adapted from Freitag, 2010; Freitag et al., 2007; Table 1).

All participants received payment for their participation. The study was approved by the Ethics Committee of the Medical Faculty at the University Leipzig, Germany (299‐12‐14092012). All participants gave written informed consent in accordance with procedures approved by the Research Ethics Committee of the University of Leipzig.

### 2.2 Experiments

**FIGURE 1:**
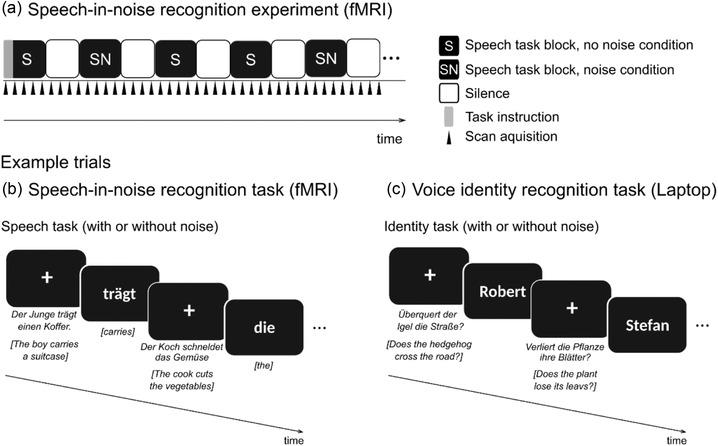
Experimental design and example trials for the speech‐in‐noise recognition experiment (a,b) and the voice identity recognition experiment (c). In both experiments, stimuli consisted of blocks of auditory sentences presented with or without background noise (noise/no noise condition). Each condition was presented in separate blocks. (a) During the speech‐in‐noise recognition experiment, fMRI volumes were acquired continuously. (b) Example trials for the speech‐in‐noise recognition experiment. Participants heard a sentence over headphones followed by a written word on the screen. Participants decided whether they had heard the word presented on the screen in the previously presented sentence or not. (c) The voice identity recognition experiment had the same design as the speech‐in‐noise recognition experiment. Participants listened to sentences spoken by the speakers from the speech‐in‐noise experiment and decided whether the written name (presented on the computer screen) matched the name of the speaker of the previously presented sentence or not.

To investigate neural responses to speech‐in‐noise, participants performed an auditory‐only speech recognition task for speech that was either presented with or without additional noise during MRI (speech‐in‐noise recognition experiment, Figure 1a,b). Design and raw data for the speech‐in‐noise recognition experiment are the same as described in Schelinski et al. (2022). For participants who never had MRI before, we conducted a mock MRI to familiarize the participants with the MRI environment. Before the speech‐in‐noise recognition experiment, participants were familiarized with the voices of the speakers presented in the experiment (speaker familiarization and voice identity recognition task; Figure 1c) and the task (task familiarization). We used Presentation software (Neurobehavioral Systems Inc., USA) to present stimuli and record responses. We presented stimuli during the fMRI experiments using an MR confon system (Mark II; MR confon, Germany). Participants wore ear plugs in addition to MRI‐compatible headphones. All stimuli in the familiarization phases (i.e., speaker and task familiarization) and the voice identity recognition experiment were presented on a laptop outside the MRI‐scanner room via headphones (HD 201, Sennheiser, Germany) and were not used during the fMRI experiments. Initial sound levels were the default values of the MR confon system, similar for all participants and adjusted to a comfortable hearing level if needed.

#### 2.2.1 Speech‐in‐noise recognition experiment

##### Stimuli

Stimuli consisted of auditory‐only five‐word sentences spoken by six male native German speakers of similar age (age range 25–31 years old). The sentences were semantically neutral, phonologically and syntactically homogeneous (e.g., “Der Junge trägt einen Koffer” [“The boy carries a suitcase”] or “Der Koch schneidet das Gemüse” [“The cook cuts the vegetables”]) and spoken in a neutral manner. All speakers were unfamiliar to all participants. The final stimulus set included 90 sentences for each speaker (45 stimuli without and 45 stimuli combined with noise). We generated auditory stimuli from audio‐visual recordings which were made from each speaker using a digital video camera (HD‐Camcorder LEGRIA HSF100; Canon Inc., Tokyo, Japan) with an external directional microphone (Sennheiser Kondensator M. System K6; Sennheiser, Wedemark, German; 44100 kHz sampling rate, 16‐bit resolution). The raw audio material was preselected using Audacity software (version 1.3.5 beta, http://audacity.sourceforg.net). For further processing of the audio material, we used Matlab software (version 8.2, The MathWorks, Inc, Natick, Massachusetts, USA). All audio stimuli were cut to have 50 ms of silence at onset and 150 ms of silence at offset. The audio stimuli from all six speakers were adjusted to the same root mean square (rms = 0.0765). To create an additional set of stimuli for the noise condition, the same audio stimuli were combined with pink noise (signal‐to‐noise ratio of −8 dB; linear 10 ms fade‐in and fade‐out).

##### Experimental design

Before the fMRI experiment, the participants were familiarized with the speakers and the task. For a detailed description, see Supporting Information. During the fMRI, participants performed speech recognition tasks on speech that was presented with additional noise (noise condition) or without (no noise condition) (Figure 1a). Each condition was presented in 18 blocks so that the experiment had 36 blocks in total. In each block, nine sentences were spoken by three of the previously familiarized six speakers (see Section "Stimuli"; 324 trials in total). At the end of each sentence, a written word (target word) appeared on the screen and the participants had to decide whether this word appeared within the sentence or not (Figure 1b). The written word was presented for 1 s. To avoid training effects for the sentences, different sentences were presented during the noise and the no noise condition. Whether a sentence was presented with or without noise was counterbalanced across subjects. All sentences were repeated for a maximum of two times within one condition. If sentences were repeated within one condition, they were spoken by a different speaker. Between blocks, there was a silent period of 18 s in which we presented a fixation cross on the screen (Figure 1a). Including the silence period, the duration of one block was approximately 45 s. Sentences within each block were presented randomly ordered. The order of blocks was presented randomly for each participant, but was the same for each matched pair of ASD and TD group participants. Responses were made via a button box using the target and the middle finger of the dominant hand. Total MRI acquisition time was approximately 27 min.

#### 2.2.2 Voice identity recognition experiment

To test voice identity recognition abilities, the participants performed a voice identity recognition task for speakers learned during the speaker familiarization phase.

##### Stimuli

Stimuli of the voice identity recognition experiment consisted of an additional set of auditory‐only five‐word sentences spoken by the same six speakers as described for the speech‐in‐noise recognition experiment (18 sentences per speaker). All sentences were semantically neutral, phonologically and syntactically homogeneous five‐word questions (e.g., “Überquert der Igel die Straße?” [“Is the hedgehog crossing the street?”]) or (e.g., “Verliert die Pflanze ihre Blätter?” [“Is the plant losing its leaves?”]). Stimuli were recorded and processed as described for the speech‐in‐noise recognition experiment.

##### Experimental design

The participants performed voice identity recognition tasks on speech that was presented with additional noise (noise condition) or without (no noise condition) (Figure 1c). Each condition was presented in 6 blocks (12 blocks in total). Each block contained 9 trials (108 trials in total). Each speaker was presented 18 times (nine trials in the noise and nine trials in the no noise condition). Sentences were different for the noise and the no noise condition. In each trial, one sentence spoken by one of the six speakers was presented. At the end of the sentence, a written name of one of the six speakers appeared on the screen. Participants were instructed to decide after each sentence, whether the sentence was spoken by the person or not. Before the start of the actual voice identity recognition experiment, participants were familiarized with the noise signal (i.e., 3 s presentation of the pure noise signal) and performed an example block which included one sentence per speaker.

### 2.3 Image acquisition

Structural T1‐weighted and functional images were acquired on a 3T Siemens Magnetom Verio scanner (Siemens, Germany) with a 32‐channel head coil for the structural and a 12‐channel head coil for the functional images.

#### 2.3.1 Functional MRI

Volumes were acquired continuously (TR = 2.81 s; 581 volumes for each participant). fMRI images were acquired using a gradient‐echo EPI (echo planar imaging) pulse sequence (TE = 30 ms; flip angle = 90°; FoV = 192 mm × 192 mm; 2 mm slice thickness; interslice gap = 1 mm resulting in a resolution of 3 mm isotropic; 42 axial slices; acquisition bandwidth = 1954 Hz; whole brain coverage; ascending acquisition). A pair of 2D gradient echo images with different echo times (TE1/TE2 = 4.92 ms/7.38 ms) was obtained for B0 field mapping (Jezzard & Balaban, 1995). These images were measured at the same slice locations as in the fMRI acquisition. Voxel resolution and image size were the same. Scanning parameters were: TR = 488 ms, flip angle 60°, pixel bandwidth = 327 Hz/pixel. Images were acquired AC‐PC oriented.

#### 2.3.2 Structural MRI

Anatomical images were acquired using a 32‐channel head coil and a T1‐weighted 3D magnetization‐prepared rapid gradient echo sequence (Mugler & Brookeman, 1990) (TR = 2300 ms; TE = 2.98 ms; TI = 900 ms; flip angle = 9°; FOV = 256 mm × 240 mm; voxel size = 1 mm^3^ [isotropic resolution]; 176 sagittal slices) with nonselective excitation and linear phase encoding. Magnetization preparation consisted of a nonselective inversion pulse. For one participant, we used a 12‐channel head coil (with an identical scanning protocol), as the 32‐channel head coil was too tight for the participants' head size. Scanning time for the structural scan was 9 min 14 s.

### 2.4 Data analysis

If not otherwise stated, all analyses included data from 17 participants with ASD and their respective matched TD group participants. Data from one ASD participant were not available for the voice identity recognition task due to technical reasons. To ensure pairwise comparison, we also excluded the data from the matched TD‐group participant for the voice identity recognition experiment (i.e., *n* = 16 for both groups).

#### 2.4.1 Behavioral data

For analyzing behavioral data, we used SPSS software (version 24, IBM SPSS Statistics, NY, USA). We used R (RCoreTeam, 2021) for creating figures. As dependent variables, we used the average percentage and reaction times of correct and incorrect responses. For recognition accuracy, we treated missed responses as incorrect responses. For group comparisons, we used analysis of variance (ANOVAs) and independent *t*‐tests. We used paired samples *t*‐tests for within‐group comparisons. All statistical tests were calculated two tailed. The level of significance was defined at *α* = .05.

#### 2.4.2 MRI data

We analyzed MRI data using standard procedures in SPM software (version 12, Wellcome Trust Centre for Neuroimaging, UCL, London, UK) in a Matlab environment (version 9.3, The MathWorks, Inc, Natick, Massachusetts, USA). For pre‐processing, images were realigned and unwrapped. Anatomical scans were co‐registered to the mean of the functional scans. Images were normalized to the Montreal Neurological Institute (MNI) standard stereotactic space and spatially smoothed with a Gaussian kernel of 4 mm full width at half maximum. For all analyses, statistical parametric maps were generated by modeling the evoked hemodynamic response for the different conditions as boxcar functions convolved with a synthetic hemodynamic response function using the general linear model (Friston et al., 2007) (high‐pass filter 128 s). We modeled the conditions “noise” and “no noise” at the first level. To account for differences in task difficulty, we included the individual differences in task performance (percent correct) between the noise and the no noise condition as covariate of no interest for within and between group task comparisons. We performed one‐sample *t*‐tests across the single‐subject contrast images for within group analyses. For between group analyses, we used two‐sample *t*‐tests comparing the means of the single‐subject contrast images from both groups.

##### Regions of interest

We focused on brain regions that have been identified to play a role in speech‐in‐noise processing in a meta‐analysis of neuroimaging studies (Alain et al., 2018) (Figure S1). These regions are the left IFG, the right insula, and the left IPL. For the region of interest (ROI) analysis, we created spheres with a radius of 5 mm surrounding the peak coordinates provided for each of these regions in the meta‐analysis (Talairach coordinates: left IFG: *x* = −36, *y* = 19, *z* = 8 [MNI space: *x* = −37, *y* = 22, *z* = 6]; right insula: *x* = 31, *y* = 18, *z* = 12 [MNI space: *x* = 32, *y* = 20, *z* = 10]; left IPL: *x* = −35, *y* = −50, *z* = 36 [MNI space: *x* = −35, *y* = −53, *z* = 38]). Transformation from Talairach space into MNI space is based on a nonlinear registration between the Talairach and the MNI brain (Lacadie et al., 2008).

##### Significance thresholds for fMRI data

We considered effects as significant at *p* < .05 family wise error corrected (FWE at peak level) for the ROI and Bonferroni corrected for the three ROIs (*p* < .016 FWE corrected). We considered effects for which we did not have an a priori hypothesis as significant at *p* < .05 FWE corrected for the whole brain.

## 3 Results

### 3.1 Behavior

#### 3.1.1 Speech‐in‐noise recognition experiment

##### Recognition accuracy

**FIGURE 2:**
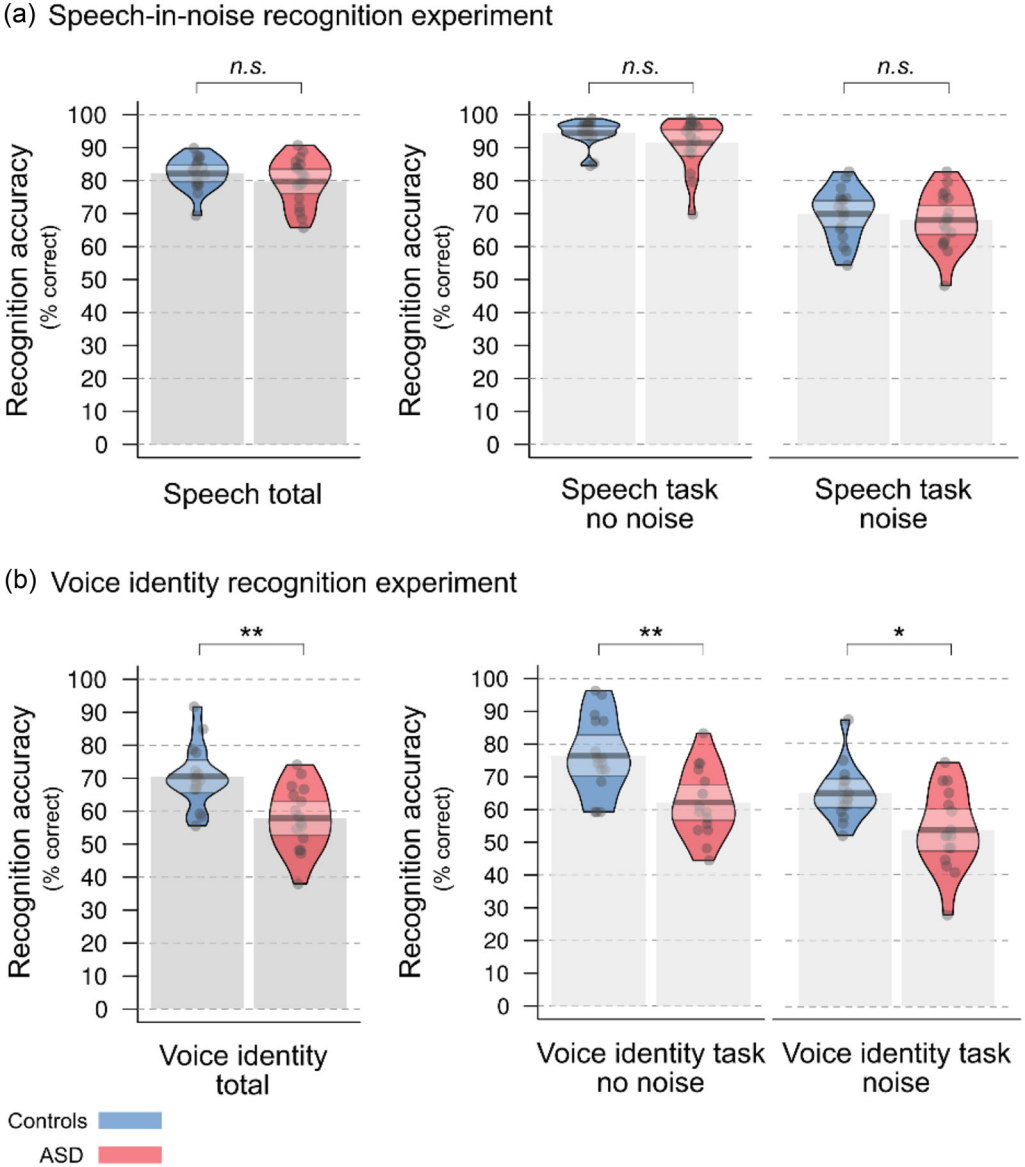
Performance accuracy in the speech‐in‐noise recognition and the voice identity recognition experiment. (a) In the speech‐in‐noise recognition experiment, there were no significant differences between the ASD and the control group. Both groups performed significantly worse in the noise condition as compared to the no noise speech task condition. (b) In the voice identity recognition experiment, the ASD group performed significantly worse as compared to the control group in the noise and the no noise condition. Both groups performed significantly worse in the noise as compared to the no noise condition. Bars represent the mean average accuracy score for each group. Dots represent mean performances from each participant. Beans represent the smoothed density curve showing the full data distribution. Bands represent the 95% confidence interval around the mean. * *p* < .05; ** *p* < .005; n.s., not significant.

For recognition accuracy, a repeated‐measures ANOVA with the between‐subject factor “group” (TD, ASD) and the within‐subject factor “noise condition” (no noise, noise) revealed no significant main effect of group and no significant interaction between the factors task and group (*p*s > .2). There was a significant main effect of noise condition (*F*(1,32) = 333.668, *p* < .001, *η^2^_p_* = 0.912) (*n* = 17 for the ASD and the control group) (Figure 2a; Table 2). A post hoc paired *t*‐test indicated that over all participants, performance was lower in the noise as compared to the no noise condition (*t*(33) = 18.493, *p* < .001, *d* = 3.172) (Figure 2a; Table 2).

**TABLE 2:** Summary of average recognition accuracy (in % correct) and reaction times (RT in ms) for the speech‐in‐noise recognition and the voice identity recognition experiment

		ASD		Controls		Group comparison	
		M	SD	M	SD	*p*	*d (r)*
**Speech‐in‐noise recognition experiment** (*n*_ASD_ = 17, *n*_Controls_ = 17)							
Recognition accuracy (in %)							
	No noise condition	91.43	7.86	94.44	3.98	.168	0.483 (0.24)
	Noise condition	68.08	8.74	69.93	7.86	.521	0.223 (0.11)
	Total	79.76	7.34	82.19	4.99	.267	0.387 (0.19)
Reaction times (in ms)							
	No noise condition	673.20	66.60	671.47	46.51	.931	0.030 (0.02)
	Noise condition	691.69	50.45	694.72	55.21	.868	0.057 (0.03)
	Total	683.68	52.08	681.82	44.20	.911	0.039 (0.02)
**Voice identity recognition experiment** (*n*_ASD_ = 16, *n*_Controls_ = 16)							
Recognition accuracy (in %)							
	No noise condition	62.15	10.41	76.43	11.89	.001*	1.278 (0.54)
	Noise condition	53.59	12.07	64.74	8.29	.005*	1.077 (0.47)
	Total	57.87	9.82	70.58	9.38	.001*	1.324 (0.55)
Reaction times (in ms)							
	No noise condition	643.33	111.47	615.96	78.48	.499	1.164 (0.50)
	Noise condition	571.84	143.86	647.67	86.15	.088	0.640 (0.31)
	Total	607.52	121.15	632.22	71.39	.439	0.248 (0.12)

*Note*: Scores are summarized as average over group with standard deviation (SD) and *p*‐values and effect sizes (Cohen's *d*) from independent *t*‐tests.

Abbreviations: M, mean; SD, standard deviation.

* Significant group differences (*p* < .05).

##### Reaction times

For reaction times, a repeated‐measures ANOVA with the between‐subject factor “group” (TD, ASD) and the within‐subject factor “noise condition” (no noise, noise) revealed no significant main effect of group and no interaction between the factors task and group (*p*s > .8). This indicates that the control and the ASD group showed similar reaction times for the task and both conditions. There was a significant main effect of noise condition (*F*(1,32) = 4.926, *p* = .034, *η^2^_p_* = 0.133) (Table 2). A post hoc paired *t*‐test indicated that over all participants, reaction times were higher in the noise as compared to the no noise condition (*t*(33) = 2.254, *p* = .031, *d* = 0.386).

#### 3.1.2 Voice identity recognition experiment

##### Recognition accuracy

The ASD group (*n* = 16) showed significantly lower performance in voice identity recognition as compared to the control group (*n* = 16) when the voice was presented with noise as well as when the speech signal was presented without noise (Figure 2b; Table 2). This was revealed by a repeated‐measures ANOVA with the between‐subjects factor “group” (TD, ASD) and the within‐subject factor “noise condition” (no noise, noise) and post hoc *t*‐tests. There were significant main effects of group (*F*(1,30) = 14.024, *p* = .001, *η^2^_p_* = 0.319) and noise condition (*F*(1,30) = 34.5444, *p* < .001, *η^2^_p_* = 0.535). There was no significant interaction between the group x noise condition (*p* = .372). Post hoc *t*‐tests indicate that the ASD group performed significantly worse in voice identity recognition as compared to the control group in both noise conditions (independent *t*‐tests: no noise: *t*(30) = 3.615, *p* = .001, *d* = 1.078; noise: *t*(30) = 3.047, *p* = .005, *d* = 1.324) (Figure 2b; Table 2) and that over all participants, performance was significantly worse in the noise as compared to the no noise condition (paired *t*‐test: *t*(31) = 5.894, *p* < .001, *d* = 1.042).

##### Reaction times

A repeated‐measures ANOVA with the between‐subjects factor “group” (TD, ASD) and the within‐subject factor “noise condition” (no noise, noise) for reaction times revealed a significant interaction (*F*(1,29) = 11.499, *p* = .002, *η^2^_p_* = 0.284). Post hoc *t*‐tests indicate that this interaction was driven by lower reaction times (i.e., faster responses) in the noise as compared to the no noise condition in the ASD group (paired *t*‐test: *t*(15) = −3.283, *p* = .005, *d* = 2,207) (Table 2). This result was unexpected. There were no significant main effects of group (*p* = .507) or noise condition (*p* = .201). These results indicate that the control and the ASD group had similar reaction times for voice identity recognition and for both noise conditions.

### 3.2 fMRI—speech‐in‐noise recognition experiment

#### 3.2.1 ROI analysis

**FIGURE 3:**
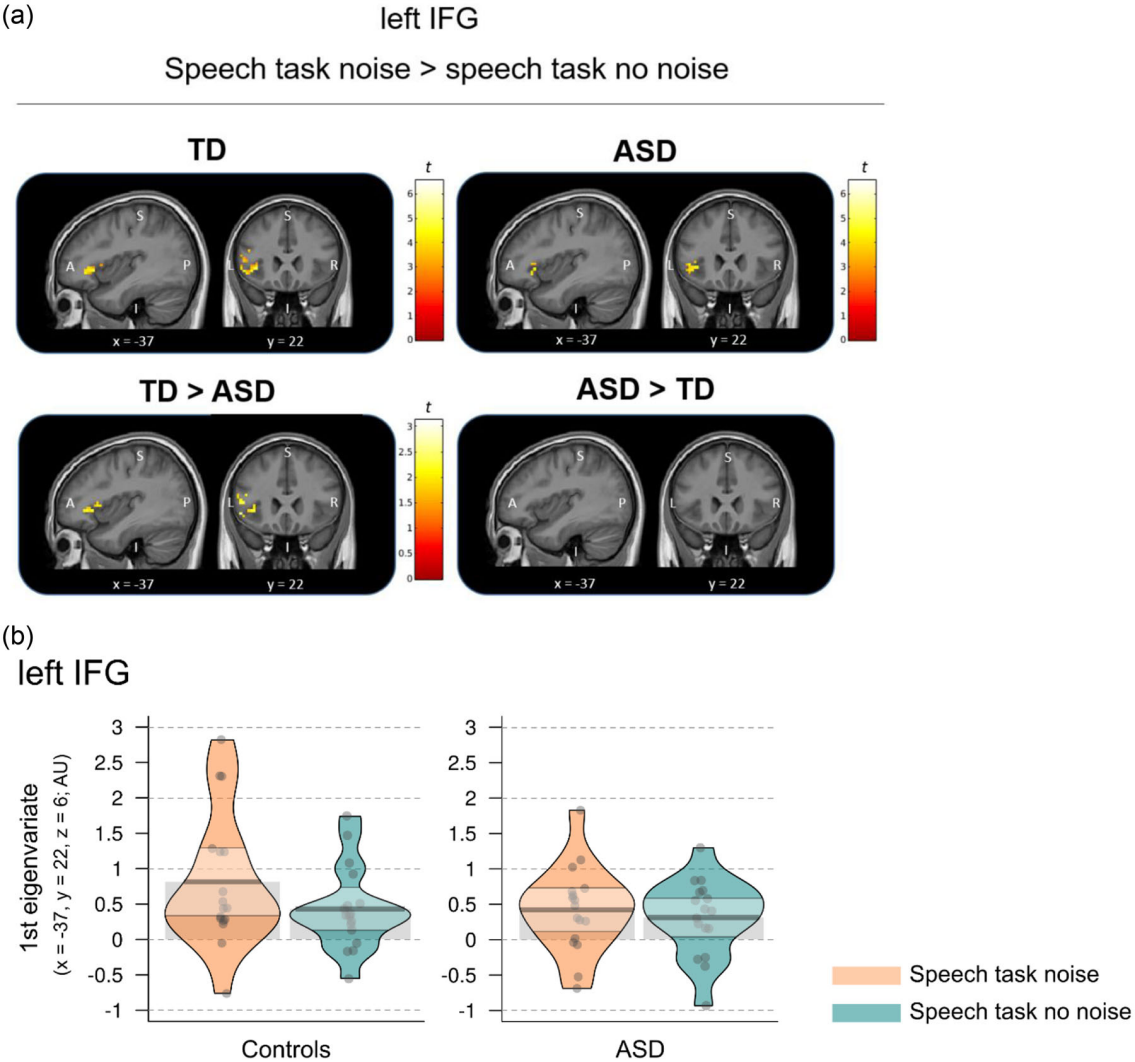
fMRI results for the ROI analyses for the left IFG. (a) The left IFG showed enhanced response for the noise as compared to the no noise condition in both groups. This enhanced response in the left IFG for the contrast noise > no noise was significantly higher in the control group as compared to the ASD group. Results are significant at *p* = .05 FWE corrected and Bonferroni corrected for 3 ROIs (i.e., left IFG, right insula, and left IPL). For display purposes only, within group results are displayed at *p* = .005 uncorrected and the interaction (TD > ASD for noise > no noise) at *p* = .05 uncorrected. Results are plotted on a group mean structural image using masks for the left IFG. Color bars represent *t*‐values. A, anterior; I, inferior; P, posterior; S, superior; L, left; R, right; *x, y* = coordinates in MNI space. (b) Parameter estimates extracted at the statistical maxima for the left IFG ROI (MNI‐coordinate: *x* = −37, *y* = 22, *z* = 6) for the interaction between group (controls, ASD) and task condition (noise, no noise). Both groups showed enhanced blood oxygenation level dependent (BOLD) responses in the left IFG for the speech task when the speech signal was presented with noise (noise condition) and when the speech signal was presented without additional noise (no noise condition). Dots represent mean performances from each participant. Beans represent the smoothed density curve showing the full data distribution. Bands represent the 95% confidence interval around the mean.

The TD group (*n* = 17) and the ASD group (*n* = 17) showed significantly higher blood‐oxygenation‐level‐dependent (BOLD) responses in the left IFG when performing the noise as compared to the no noise task condition (Figure 3a; Table 3). There was an interaction between noise condition and group, indicating that responses in the left IFG for the contrast “noise > no noise” were significantly higher in the TD group as compared to the ASD group (*p* = .010 FWE corrected and Bonferroni corrected for the three ROIs) (Figure 3). For the same contrast (i.e., “noise > no noise”), there were no significantly higher responses in the right insula or the left IPL within or between the groups. Both groups had significant BOLD responses in the left IFG and the right insula for the main effects noise and no noise and there were no significant group differences for these responses (Figure 3b; Table 3). For the same main effects (i.e., “noise,” “no noise”), there were enhanced responses in the left IPL within the TD group. For the left IPL, there was also a detectable enhanced response for the noise condition in the ASD group; however, this response did not survive the Bonferroni‐correction for the three ROIs (*p* = .016 FWE corrected). Responses in the left IPL were not significantly different between the TD and the ASD group for the noise or no noise main effect.

**TABLE 3:** Coordinates for significant BOLD‐responses for the ROI analysis (*p* < .016 FWE‐corrected at peak level and Bonferroni corrected for three regions of interest)

	**Speech task noise**							
	Control group				ASD group			
	*x*	*y*	*z*	*Z*	*x*	*y*	*z*	Z
Left IFG	−33	23	2	4.58	−33	23	5	3.68
Right Insula	33	23	8	3.58	30	23	10	3.80
Left IPL	−33	−52	41	3.34	−33	−52	42	3.21
					*−33*	*−49*	*36*	*2.73*
	Controls > ASD				ASD > Controls			

	**Speech task no noise**							
	Control group				ASD group			
	*x*	*y*	*z*	*Z*	*x*	*y*	*z*	*Z*
Left IFG	−33	23	2	3.72	−33	23	2	3.96
	−*39*	*20*	*5*	*2.74*				
Right Insula	33	20	8	3.69	30	23	11	3.74
Left IPL	−33	−52	41	3.32		–		
	Controls > ASD				ASD > Controls			

	**Speech task noise > no noise**							
	Control group				ASD group			
	*x*	*y*	*z*	*Z*	*x*	*y*	*z*	*Z*
Left IFG	−33	23	2	4.21	−33	26	5	3.61
	Controls > ASD				ASD > Controls			
Left IFG	−33	23	5	3.28		–		

*Note*: Italic coordinates indicate peaks of sub‐clusters within a significant cluster.

Abbreviations: IFG, inferior frontal gyrus; IPL, inferior parietal lobule; *x, y, z*, peak coordinates in MNI space (in mm).

#### 3.2.2 Whole brain analysis

**FIGURE 4:**
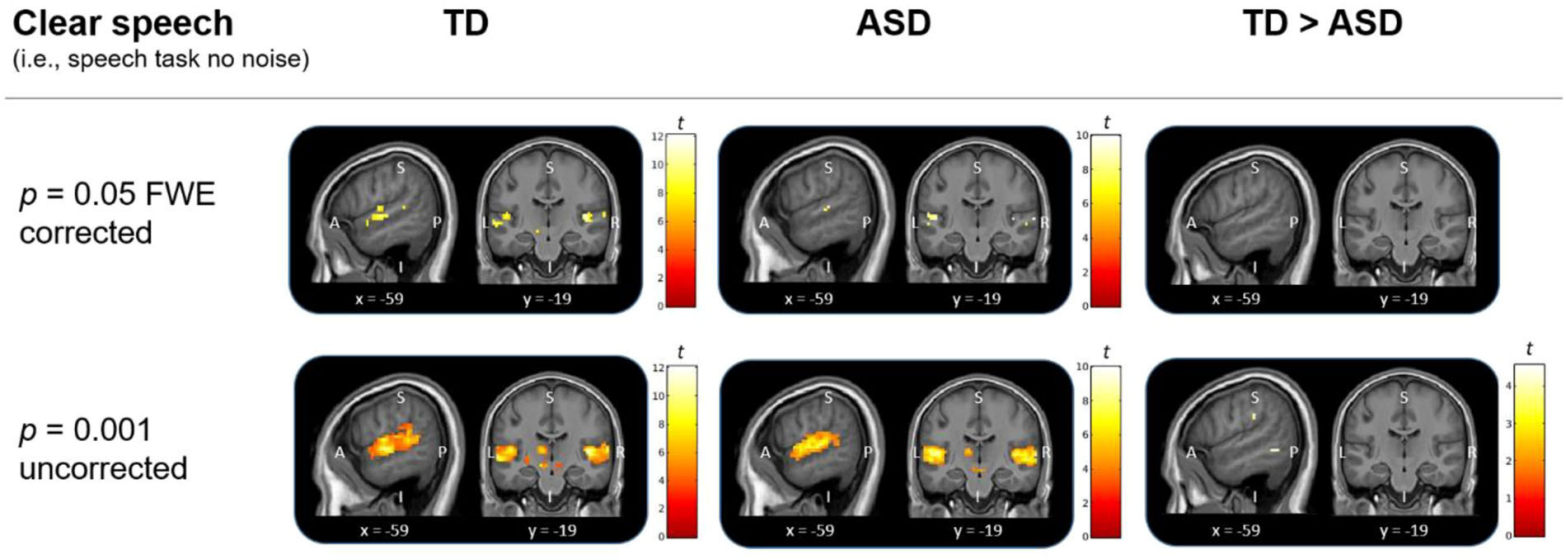
Whole brain fMRI responses to clear speech (i.e., speech task no noise condition). The TD and the ASD group showed similar responses in speech‐associated areas including bilateral Heschl's gyrus and STS/G (*p* = .05 FWE corrected for whole brain). Results are visualized at *p* = .05 FWE corrected and for information purposes only additionally at *p* = .001 uncorrected. At this lenient threshold, there are clusters in the medial temporal gyrus, supramarginal gyrus, and the frontal pole, with a volume of < 5 voxels. Results are plotted on a group mean structural image using implicit masks for the left IFG. Color bars represent *t*‐values. A, anterior; I, inferior; P, posterior; S, superior; L, left; R, right; *x*, *y* = coordinates in MNI space.

In a previous study, we showed that cerebral cortex responses to clear speech are comparable between typically developed individuals and individuals with ASD (Schelinski et al., 2016). Here, we replicated this finding: For the clear speech condition (i.e., main effect no noise), both the TD and the ASD group showed very similar responses (Figure 4). For a full list of regions, see Table S2. There were no significant differences between the two groups in any brain region for the clear speech condition (*p* > 0.05 FWE corrected for the whole brain). For information purposes only, we additionally report whole brain results for the clear speech and the noise condition at a lenient level of significance (*p* < .001 uncorrected; Tables S3 and S4).

#### 3.2.3 Head motion during fMRI

The ASD and the TD group did not differ significantly in the average amount of head movements (all *p* values > .2; Table S5).

## 4 DISCUSSION

There are three key findings from our study on speech‐in‐noise processing in ASD. First, for speech‐in‐noise recognition as compared to speech recognition without noise (clear speech), the left IFG responded less in the ASD group as compared to the control group. Second, the ASD group showed typical cerebral cortex responses when processing clear speech. Third, replicating our previous behavioral findings, for clear speech, the ASD group performed worse as compared to the control group in voice identity recognition, whereas both groups performed comparable in speech recognition. The ASD group also had lower recognition accuracy for identity recognition when the voice signal was presented with noise. Unexpectedly, there were no significant group differences for performance in the speech‐in‐noise recognition task.

We provide novel evidence that processes related to speech‐in‐noise recognition in ASD are particularly altered in the left IFG. Only a few studies investigated the neural processing of speech‐in‐noise in ASD (Hernandez et al., 2020; Lin et al., 2021; Russo et al., 2009; Schelinski et al., 2022). Russo et al. (2009) found altered brainstem responses in children with ASD as compared to typically developing children when passively listening to speech (i.e., syllables) when the speech signal was presented with and without additional noise (i.e., presented together with white noise). In a recent study, Hernandez et al. (2020) showed increased cerebral cortex responses in adolescents with ASD as compared to typically developing controls when passively listening to a conversation of two people when the conversation was presented with as compared to when the conversation was presented without additional noise (i.e., noises from everyday life, such as a police siren). This increased response in the left angular gyrus was interpreted as compensatory. The left angular gyrus is part of the left IPL (Igelstrom & Graziano, 2017) and has been found to play a causal role in facilitating speech‐in‐noise recognition (Hartwigsen et al., 2015). In our study, we did not find such enhanced response in another part of the left IPL, neither within the control nor within the ASD group. Contrasting the results from Hernandez et al. (2020), Lin et al. (2021) found evidence for reduced cortical connectivity between the left temporoparietal junction (TPJ) and the left dorsal premotor cortex in adults with ASD as compared to controls when participants were asked to attentively listen to (i.e., indicate if a sentence was intelligible or not) spectrally degraded speech (i.e., noise vocoded speech) as compared to when listening to clear speech and spectrally rotated unintelligible speech. The TPJ is a brain area that overlaps with the IPL at the intersection of the angular gyrus, supramarginal gyrus, and the posterior superior temporal lobe (Igelstrom & Graziano, 2017). In these previous studies, participants passively listened to the auditory stimuli (Hernandez et al., 2020; Russo et al., 2009) or attention was attempted to be increased by an intelligibility task (Lin et al., 2021). However, since all these three studies did not include an explicit task to recognize speech, they cannot address brain responses when recognizing speech‐in‐noise. This point is critical, because differences in task instruction in speech‐in‐noise perception lead to the recruitment of different brain mechanism (Wild et al., 2012). Evidence that neural processing is altered when performing a speech recognition task comes from a previous study which included the same ASD and TD group participants as reported here (Schelinski et al., 2022). In that study, the TD group but not the ASD group showed enhanced responses in the right IC for speech‐in‐noise recognition as compared to speech recognition without noise. However, IC‐responses were not significantly different between the two groups (Schelinski et al., 2022). Here, we provide evidence that the cerebral cortex processing of speech‐in‐noise is altered in adult ASD as compared to typically developed adults. Our study rests on a sample (*n* = 34) that is relatively homogeneous (i.e., adults with an IQ and verbal abilities at least within the normal range). This means that we do not know whether similar alterations as found in the present study can also be found in larger and more heterogeneous samples representing the whole autism spectrum.

Our findings suggest that in ASD, the recognition of speech under noisy listening conditions is particularly reduced in the left IFG. The peak coordinate for the left IFG found in the meta‐analyses by Alain et al. (*x* = −37, *y* = 22, *z* = 6) for speech‐in‐noise recognition is in close proximity to the left insula (compare, e.g., Smith et al., 2004). A number of previous studies showed that the left IFG and insula are involved in challenging listening conditions (e.g., Davis et al., 2011; Davis & Johnsrude, 2003; Giraud et al., 2004; Golestani et al., 2013; Obleser et al., 2007). Comparing brain responsiveness to different levels of distortion of the speech signal, the greatest involvement of the left IFG was observed when the speech signal was more difficult to understand but still intelligible as compared to when the speech signal was clear speech or unintelligible due to distortion (Davis et al., 2011; Davis & Johnsrude, 2003; Du et al., 2014; Golestani et al., 2013; Peelle et al., 2010). This fits well with our results where both groups performed significantly worse in the speech‐in‐noise condition as compared to the no noise condition but the speech signal was still intelligible as indicated by performance well above chance in both groups for the speech‐in‐noise condition (i.e., correct responses of about 70% in the control group and 68% in the ASD group).

The left IFG and adjacent areas (including the left insula) are integral parts of both the speech perception as well as the speech production network (Friederici, 2012; Hickok & Poeppel, 2007; Price, 2012). One line of research highlights the supportive role of sensorimotor brain regions (including the left IFG and insula) for speech perception (e.g., Davis & Johnsrude, 2007; Hickok et al., 2011; Iacoboni, 2008; Pulvermuller et al., 2006; Rauschecker & Scott, 2009; Wilson et al., 2004). Recent studies in typically developed adults suggest that such a supportive role of motoric brain regions, including the left IFG and adjacent areas (i.e., insula), is particularly important in speech‐in‐noise perception (Adank et al., 2012; Binder et al., 2004; Du et al., 2014; Hervais‐Adelman et al., 2012; Shahin et al., 2009; Wild et al., 2012; Zekveld et al., 2006). For example, in a previous study, enhanced responses in the left IFG/insula were associated with the recognition of noise vocoded single words (Hervais‐Adelman et al., 2012). In another study, activity of the left IFG increased with decreasing SNRs, indicating higher responses of the left IFG when the speech signal (i.e., syllables added with white noise) was more difficult to understand (Binder et al., 2004). A similar mechanism is assumed for the supportive role of sensory‐motor regions when comprehending speech‐in‐noise across studies: The incoming speech input is compared against articulatory representations or templates (hold in the sensory‐motor system) and is simulated via these sensory‐motor regions which helps to predict what is said and thus facilitates speech comprehension (Binder et al., 2004; Du et al., 2014; Hervais‐Adelman et al., 2012; Shahin et al., 2009; Zekveld et al., 2006). This mechanism might be particularly important when speech is more unpredictable, which requires more error correction (Du et al., 2014). In a predictive coding view, such a mechanism would assume that we have a generative model of the speaker's speech signal (von Kriegstein et al., 2008). By simulating the articulatory movements of a speaker, we can better predict what the speaker is saying, particularly in noisy listening conditions. We suggest that differences in this mechanism can explain group differences in the left IFG/insula. More specifically, we assume that enhanced activity in the left IFG/insula in response to speech‐in‐noise as compared to clear speech in the control group could be explained by a facilitative effect of sensory‐motor representations which simulate the incoming speech input. In contrast, for the ASD group, we suggest that this mechanism is less pronounced and which leads to a less pronounced facilitation of speech perception in challenging listening conditions.

The IFG has been associated with further top‐down functions which may be supportive of recognizing speech in challenging listening conditions, such as attention (Wild et al., 2012), verbal working memory (e.g., Du et al., 2014; Zekveld et al., 2006), and semantic and syntactic integration (e.g., Davis et al., 2011). In line with findings by Wild et al. (2012), group differences in our study could also be explained by a top‐down effect of attention. Wild et al. (2012) found enhanced left IFG responses for attended degraded speech (i.e., noise‐vocoded sentences) as compared to ignored speech or clear speech. In this sense, the processing of degraded speech could be also enhanced by attention and engaging higher‐order mechanisms that modulate early perceptual processing, a mechanism which could be more pronounced in the TD as compared to the ASD group. We assume that our results are unlikely to be explainable by group differences in verbal working memory, because both groups were matched on verbal working memory and did also not differ in verbal‐IQ (Table 1). We further assume that group differences in the left IFG were not primarily due to differences in semantic or syntactic processing, since sentences in both task conditions (noise and no noise) had the same structure and were syntactically homogenous and semantically neutral. Additionally, whether specific sentences of the stimulus set were presented with or without noise was counterbalanced over participants, but the same for each TD‐ASD pair. Further, our results are unlikely to be explained by behavioral group differences, since there were no significant group differences for the no noise or the noise condition.

The ASD group showed typical neural processing for clear speech in brain areas associated with clear speech processing, including bilateral Heschl's gyrus and superior temporal sulcus and gyrus (STS/G) (Friederici, 2012). This is in line with recent results from a meta‐analysis on speech perception in ASD which showed mostly similar cerebral cortex responses when processing clear speech in ASD and typically developed individuals including bilateral STS/G and left IFG (Tryfon et al., 2018). Further, our results replicate previous study findings in which we showed that the neural processing of clear speech recognition is on a neurotypical level in adults with high‐functioning ASD (Schelinski et al., 2016). In that study, we found that the neural responses to speech were not significantly different between groups of people with ASD and typically developed controls when performing a speech recognition task as compared to when performing a voice identity recognition task.

The level of speech‐in‐noise recognition abilities in ASD varied between previous studies, likely due to differences in the experimental design (Alcantara et al., 2004; Foxe et al., 2015; Groen et al., 2009; Schelinski & von Kriegstein, 2020). Though we found group differences in the cerebral cortex for speech‐in‐noise processing, we did not find group differences at the behavioral level. In a previous study, we used an adaptive tracking procedure to detect individual thresholds in speech‐in‐noise recognition and found significant group differences with an even higher noise level of SNR of −8 (Schelinski & von Kriegstein, 2020). In that study, adults with ASD had higher speech reception thresholds (SRTs) (mean −7.59 dB SNR) as compared to typically developed control group participants (mean −9.04 dB SNR) for speech (i.e., two‐word sentences) that was presented in speech‐shaped‐noise. Lower thresholds in the control group indicated better speech recognition performance in higher levels of noise. A comparable speech‐in‐noise recognition performance for ASD and control group participants as observed in the present study is in line with previous studies in which both groups showed similar SRTs for speech that was presented in continuous background noise (Alcantara et al., 2004; Groen et al., 2009). Alcantara et al. (2004) found comparable SRTs between a group of adolescents and young adults with ASD when the speech stimuli (i.e., sentences including key words) were presented together with speech‐shaped noise. Similarly, Groen et al. (2009) found comparable SRTs between a group of children and adolescents with ASD and control group participants when the speech stimuli (i.e., single words) were presented together with pink noise. We assume that the difference between results can most likely be explained by the thresholds chosen for correct speech recognition performance. Alcantara et al. (2004) and Groen et al. (2009) used a threshold of 50% correct speech recognition performance to determine SRTs in an adaptive tracking design. That means that the thresholds refer to a performance where 50% of the speech could be correctly recognized. In contrast, in our previous study, we used a higher recognition criterion of 75% correct responses (Schelinski & von Kriegstein, 2020). Performance accuracy in the present study was about 69%, which is also below 75%. Neural differences found in the present study indicate that speech‐in‐noise was altered in the ASD group. However, behavioral differences might occur only with higher recognition rates (i.e., when the speech signal is less noisy and thus easier to understand for the control group). Other factors, such as differences in type of background noise, task difficulty, or task design (e.g., speech stimuli) could further contribute to the different study findings.

We replicate previous findings that voice identity recognition is impaired in ASD (Boucher et al., 1998; Klin, 1991; Schelinski et al., 2016, 2014, 2017) and that this impairment is dissociable from intact speech recognition in listening conditions when the speech signal is relatively easy to understand (i.e., presented with continuous pink noise with a relatively high SNR of +3 (Schelinski, 2014) or without additional noise during fMRI (Schelinski, 2016). Further, our results indicate that voice recognition is affected similarly for the noise and the no noise condition (i.e., there was no task × group interaction which would suggest a more pronounced impairment for the noise as compared to the no noise condition). Merging previous and our current behavioral results on voice identity and speech recognition in ASD, we suggest that voice identity recognition is more affected than speech recognition in challenging but also in normal hearing conditions. The investigation of the neural mechanisms of voice identity recognition in noise and its relation to the neural processing of speech recognition in noise is an interesting future research direction.

Noise significantly impacts everyday functioning and can restrict communication (for reviews, see Klatte et al., 2013; Picard & Bradley, 2001; Szalma & Hancock, 2011; van der Kruk et al., 2017). For example, restricted perception of what another person is saying in a noisy environment can impede a fluent social conversation and makes a conversation more challenging. Consequently, this leads to enhanced experience of stress in communication (Mackersie & Cones, 2011; McGarrigle et al., 2017). An enhanced stress experience might in turn lead to avoidance of potentially noisy situations and places which is an often‐reported phenomenon for people with ASD (Stiegler & Davis, 2010). Here, we provide evidence that difficulties in speech recognition in ASD are not related to a general dysfunction of cerebral brain regions related to basic auditory or speech processing. Our results rather indicate that reduced functioning in the left IFG—a cerebral cortex region particularly related to speech‐in‐noise perception—might be important in explaining restricted speech comprehension in noisy environments in ASD.

## AUTHOR CONTRIBUTIONS

Stefanie Schelinski and Katharina von Kriegstein designed the study. Stefanie Schelinski coordinated the study, performed the experiment, and analyzed the data. Stefanie Schelinski and Katharina von Kriegstein wrote the manuscript. All authors read and approved the final manuscript.

## CONFLICT OF INTEREST

The authors declare no conflict of interest.

### PEER REVIEW

The peer review history for this article is available at https://publons.com/publon/10.1002/brb3.2848.

## Supporting information

Supplementary Figure 1 Overview of regions of interest (ROI) masks
Supplementary Table 1 Overview of diagnostic scores in the ASD group
Supplementary Table 2 Coordinates for significant BOLD‐responses for clear speech (i.e., speech task no noise task condition > baseline) (*p* < .05 FWE‐corrected for the whole brain)
Supplementary Table 3 Overview of BOLD‐response maxima for clear speech (i.e., speech task no noise task condition > baseline) (*p* < .001 uncorrected for the whole brain) for information purposes only
Supplementary Table 4 Overview of BOLD‐response maxima for speech‐in‐noise (i.e., speech task noise task condition > baseline) (*p* < .001 uncorrected for the whole brain) for information purposes only
Supplementary Table 5 Group comparisons of the average movement (in mm) for all directions (x, y, and z)

## Data Availability

The dataset generated and/or analyzed during the current study are not publicly available due to privacy or ethical restrictions. SPM files will be publicly available after acceptance of the manuscript on OSF.
