# Supplementary material for: Responses in left inferior frontal gyrus are altered for speech‐in‐noise processing, but not for clear speech in autism
Source: Brain Behav. 2022 Dec 27;13(2):e2848. doi: 10.1002/brb3.2848 (PMC9927852; doi:10.1002/brb3.2848)
Supplement: Supplementary file 1 — Supplementary Figure 1 Overview of regions of interest (ROI) masks Supplementary Table 1 Overview of diagnostic scores in the ASD group Supplementary Table 2 Coordinates for significant BOLD‐responses for clear speech (i.e., speech task no noise task condition > baseline) (p < .05 FWE‐corrected for the whole brain) Supplementary Table 3 Overview of BOLD‐response maxima for clear speech (i.e., speech task no noise task condition > baseline) (p < .001 uncorrected for the whole brain) for information purposes only Supplementary Table 4 Overview of BOLD‐response maxima for speech‐in‐noise (i.e., speech task noise task condition > baseline) (p < .001 uncorrected for the whole brain) for information purposes only Supplementary Table 5 Group comparisons of the average movement (in mm) for all directions (x, y, and z) [file BRB3-13-e2848-s001.pdf]

Supplementary Material

Responses in left inferior frontal gyrus are altered for speech-in-noise processing, but not for clear speech in autism

Stefanie Schelinski [1,2] & Katharina von Kriegstein [1,2]

[1] Technische Universität Dresden, Faculty of Psychology

[2] Max Planck Institute for Human Cognitive and Brain Sciences

Methods

Speaker familiarisation

The six speakers presented during the speech-in-noise recognition experiment were familiarised to the participants during an audio-visual training before the actual MRI experiment. In this audio-visual training, participants learned to recognise voices, names and faces of all speakers.

Audio-visual training: The six speakers were familiarised during an audio-visual training before the speech-in-noise recognition and the voice identity recognition experiments. In this audio-visual training, participants learned to recognise voices, names and faces of all speakers. The design from the audio-visual training was adapted from previous studies in which we investigated the influence of audio-visual speaker familiarisation on auditory-only perception (Schelinski, Riedel, & von Kriegstein, 2014; von Kriegstein et al., 2008). Voices of the six speakers were learned together with a visual stimulus in two different learning conditions (see below stimuli and design description for details). The two conditions were part of a different study, which will be reported elsewhere (Schelinski & von Kriegstein, in prep.). Within the scope of the current study, we averaged the results for speech recognition for the noise and the no noise task condition over both learning conditions. There was a break of approximately 45 minutes between the audio-visual training and the actual scanning session in which participants were allowed to rest and were transferred to the MR-centre (10 min walk).

Stimuli. We used audio-visual recordings from the six speakers, which we described for the speech-in-noise recognition experiment. Stimuli used in the audio-visual training were not used during the speech-in-noise recognition or the voice identity recognition experiment. We recorded the face of each speaker in 16:9/ XP+ with a resolution of 1920 x 1080 pixels. Video recordings were edited using FinalCutPro (Video Studio). Videos were cut that each speaker

was shown 500ms before and after face-movement. Recordings were done against a uniform black background. We extracted static face images with closed mouth from each speaker from the video recordings.

Experimental design. The training consisted of at least two and maximal three learning and evaluation sessions. During the learning session, we instructed participants to memorise names and voices of six speakers. In each session, there were two learning conditions. In the 'static face learning condition', participants learned the voices and names of three of the speakers together with a static picture of their face. In the 'moving face learning condition', voices and names of the other three speakers were learned together with a video showing the speaker articulating. In one learning session, each speaker was presented for 15 times articulating 5-word sentences (15 trials x 3 speakers x 2 learning conditions = 90 trials in total). In each trial, the name of the speaker was presented for 1 sec, followed by a sentence spoken by that speaker and the simultaneously presented face (static or moving depending on the learning condition). Trials were presented in blocks of five trials in which the same speaker identity was presented (one block per speaker). In the following blocks, trials with different speaker identities were randomly intermixed. There were two sets of speakers: speaker set 1 containing speakers A, B and C and speaker set 2 containing speakers D, E and F. Half of the participants learned speaker set 1 in the 'static face learning condition' and speaker set 2 in the 'moving face learning condition'. The other half of participants learned speaker set 1 in the 'moving face learning condition' and speaker set 2 in the 'static face learning condition'. Static face learning and moving face learning were presented separately. The order of the two learning conditions was balanced over the whole sample: half of the participants started with the static face learning, whereas the other half of participants started with the moving face learning.

Evaluation. Learning success was evaluated after each learning session. Learning and evaluation session were performed twice. If the participant did not reach a criterion of 75% correct performance after the second repetition, learning and evaluation were repeated a third time. In the evaluation, the voice of one of the six speakers was presented either followed by a written name or by a picture of a face from one of the three speakers learned in the corresponding learning condition. Participants had to decide whether the voice and the face belonged to same person or not. Audio-visual feedback of the correct combination was provided immediately after each response. In this feedback, the voice was presented together with the name and the static or moving (i.e., articulating) face respectively. In total, each evaluation session included 48 trials (8 trials x 6 speakers). In half of the trials, voices were followed by a name, in the other half voices were followed by the face.

Task familiarisation

To familiarise the participants with the task and noise of the speech-in-noise experiment inside the MRI-machine, participants received the task instructions and performed example blocks before the actual MRI experiment. To ensure that all participants understood the task equally well, the task familiarisation was repeated if a participant had more than one missed response in the practise trials. Sentences presented during the task familiarisation were not presented during the test session. To familiarise participants with the noise and task inside the MRI-machine, we additionally presented two training blocks within the MRI-environment (one block for the noise and one block for no noise condition).

Supplementary Figures

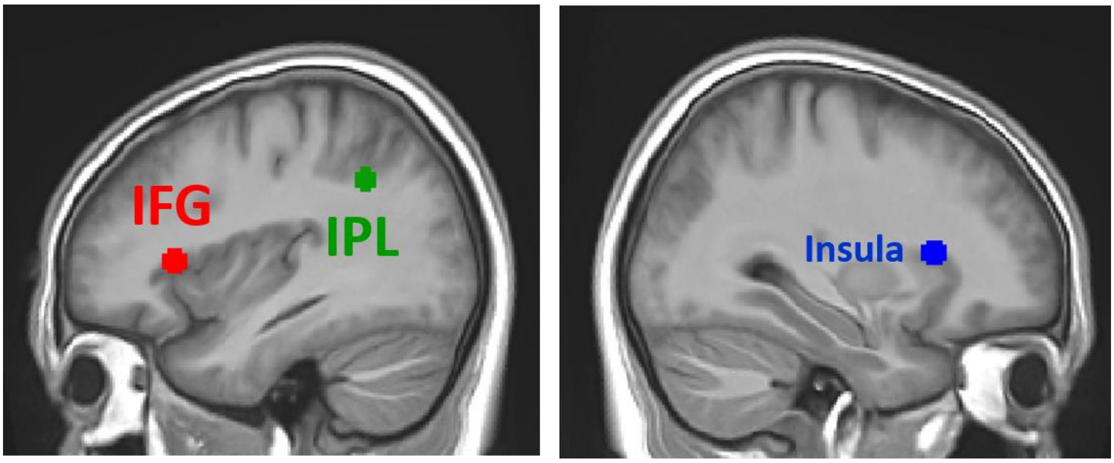

Supplementary Figure 1

Overview of regions of interest (ROI) masks. Masks are plotted on a group mean structural image. We created 5mm spheres around peak coordinates reported in a meta-analyses of neuroimaging studies investigating speech-in-noise processing, i.e., left inferior frontal gyrus (IFG, red), right insula (blue), and left inferior parietal lobule (IPL, green) (Alain, Du, Bernstein, Barten, & Banai, 2018).

Supplementary Tables

Supplementary Table 1

Overview of diagnostic scores in the ASD group.

Diagnostic test		*M* *(cut-offs for autism / autism spectrum)*	
Participants as informant			
Interview	ADOS[a] (*n* = 16)		
	Social Interaction & Communication	10.31 *(12 / 7)*	2.18
	Social Interaction	6.58 *(7 / 4)*	1.83
	Communication	3.76 *(3 / 2)*	1.13
Parents as informant			
Questionnaire	SCQ[b] (*n* = 9)	20.89 *(15)*	6.01
Interview	ADI-R[c] (*n* = 11)		
	Social Interaction & Communication	30.18	11.37
	Social Interaction	17.09 *(17)*	7.30
	Communication	12.18 *(8)*	4.81

M = mean; *SD* = standard deviation.
[a] ADOS = Autism Diagnostic Observation Schedule (Lord et al., 2000; German version: Rühl, Bölte, Feineis-Matthews, & Poustka, 2004).
[b] SCQ = Social Communication Questionnaire (Rutter, Bailey, & Lord, 2003; German version: Bölte & Poustka, 2006).
[c] ADI-R = Autism Diagnostic Interview- Revised (Lord, Rutter, & Le Couteur, 1994; German version: Bölte, Rühl, Schmötzer, & Poustka, 2003).

Supplementary Table 2

Coordinates for significant BOLD-responses for clear speech (i.e., speech task no noise task condition > baseline) ($p < 0.05$ FWE- corrected for the whole brain). Grey font = significant at peak level within the significant cluster.

	Speech task no noise											
	Control group						ASD group					
		x	y	z	*Z*	cluster size		x	y	z	*Z*	cluster size
Heschl's Gyrus TE 1.0 / TE 1.1	r	45	-19	5	6.00	64	r	42	-19	5	5.24	6
STS/STG		51	-25	5	5.65		r	42	-31	11	4.90	2
							l	-51	-22	5	5.29	32
								-45	-28	8	5.07	
								-36	-31	14	4.91	
							r	42	-19	5	5.24	6
							r	42	-31	11	4.90	2
TE 1.2		57	-7	-1	5.34		l	-54	-7	-1	4.85	4
MTG	l	-60	-13	-1	5.65	84	l	-57	-28	2	5.16	13
Heschl's Gyrus TE 1.0 / TE 1.1		-39	-25	8	5.45			-48	-31	2	5.01	
	l	-63	-37	2	5.12	5						
		-57	-40	8	4.93							
		-48	-19	8	5.44							
Cerebellum	r	12	-73	-22	5.45	19	r	27	-55	-22	4.94	1
		12	-76	-37	5.17		r	36	-61	-22	4.88	2
	r	30	-58	-28	5.35	5						
	r	21	-73	-43	4.78	1						
STS/STG	r	66	-16	8	5.36	3	r	66	-19	5	5.54	2
	r	57	-10	-7	4.86	1	r	48	-25	2	5.28	2
							r	57	-16	-1	5.20	7
	l	-57	-1	-7	4.89	2	l	-57	-19	-1	4.81	1
	l	-60	-37	14	4.86	1						
Putamen	l	-21	-4	14	4.98	4						
	l	-24	5	11	4.96	2						
Thalamus	l	-9	-16	8	5.08	2						
IPL	l	-30	-52	47	4.79	1						

	Controls > ASD	ASD > Controls
	-	-

Coordinates represent local activation maxima in MNI space (in mm) for the whole brain. Cluster size represents the number of voxels within a cluster. Clusters are reported that reached p = 0.05 FWE- corrected (peak-level). Regions were labelled based on a standard anatomical atlas implemented in SPM (SPM Anatomy Toolbox; Eickhoff et al., 2005). If not labelled in SPM or when the labelling was ambiguous, we additionally used atlases implemented in FSL (Smith et al., 2004; Harvard-Oxford cortical and subcortical structural atlases; Desikan et al., 2006). r = right; l = left; MTG = Middle Temporal Gyrus; STS/STG = Superior Temporal Sulcus/Superior Temporal Gyrus; IPL = Inferior Parietal Lobule.

Supplementary Table 3

Overview of BOLD-responses maxima for clear speech (i.e., speech task no noise task condition > baseline) (p < 0.001 uncorrected for the whole brain) for information purposes only. Grey font = significant at peak level within the significant cluster (↓ indicates that the corresponding peak-coordinate is part of the cluster that is described by the cluster peak-coordinate below).

	Speech task no noise											
	Control group						ASD group					
		x	y	z	*Z*	cluster size		x	y	z	*Z*	cluster size
Heschl's Gyrus TE 1.0 / TE 1.1	r	45	-19	5	6.00	542	↓	42	-19	5	5.54	
STS/STG		51	-25	5	5.65		r	66	-19	5	5.54	621
		66	-16	8	5.36			48	-25	2	5.23	
MTG extending to STS/STG	l	-60	-13	-1	5.65	756	↓	-57	-28	2	5.16	
STS/STG		-48	-19	8	5.41		l	-51	-22	5	5.29	698
								-45	-28	8	5.07	
Heschl's Gyrus		-39	-25	8	5.45							
IFG		-45	26	17	4.55							
		-42	20	26	4.40							
IFG	r	39	17	8	4.25	10						
	l	-51	14	8	3.40	6						
Cerebellum	r	12	-73	-22	5.45	630	r	27	-55	-22	4.94	418
		30	-58	-28	5.35			36	-61	-22	4.83	
		12	-76	-37	5.17			12	-79	-37	4.52	
	r	9	-55	-34	3.52	11						
	l	-33	-64	-25	4.64	112	l	-39	-64	-28	3.56	35
		-39	-52	-28	4.45			-27	-61	-28	3.43	
	l	-6	-55	-34	3.78	7	l	-9	-76	-31	3.77	30
		-15	-58	-37	3.38			-6	-70	-16	3.52	
Thalamus	l	-9	-16	8	5.08	204	l	-12	-16	8	4.27	25
		-21	-4	14	4.98							
Putamen		-24	5	11	4.96							
	r	12	-22	-10	4.20	19						
Brainstem	l	-12	-19	-10	4.91	61	l	-6	-34	-43	3.73	7
		-6	-28	-7	4.48		r	9	-25	-10	4.05	58

								6	-28	-19	4.00	
								3	-19	-13	3.87	
Thalamus		-18	-25	-4	4.28							
IPL	l	-30	-52	47	4.79	79	l	-27	-52	47	4.53	39
		-27	-61	50	4.19			-27	-58	32	3.27	
Putamen	r	24	8	11	4.39	94	l	-21	2	8	3.83	27
		24	11	2	4.20			-21	-4	17	3.62	
		18	-7	11	4.05							
Insula	l	-27	26	8	4.12	34	l	-30	23	2	4.04	15
		-27	20	-1	3.67							
		-27	-22	-1	3.89	14						
							r	30	23	11	3.78	8
Precentral Gyrus							l	-42	2	35	4.45	148
								-42	-4	41	3.92	
								-36	8	32	3.89	
ITG							l	-42	-49	-16	3.86	27
								-42	-55	-10	3.77	
SMA / Paracingulate Gyrus	l	-3	8	50	4.07	63	l	-6	8	53	4.53	53
		-3	8	59	4.05			9	8	53	3.53	
		9	14	50	3.83			3	14	44	3.29	
Paracingulate Gyrus	r	12	17	38	3.68	6	r	12	11	41	3.43	6
Occipital Pole	r	15	-97	-1	3.65	9						
	l	-12	-94	-4	3.48	6						
Nucleus Caudatus							r	21	5	17	3.37	7

Coordinates represent local activation maxima in MNI space (in mm) for the whole brain. Cluster size represents the number of voxels within a cluster. Clusters are reported that reached p = 0.001 uncorrected (peak-level) and a cluster size > 5 voxels. Regions were labelled based on a standard anatomical atlas implemented in SPM (SPM Anatomy Toolbox; Eickhoff et al., 2005). If not labelled in SPM or when the labelling was ambiguous, we additionally used atlases implemented in FSL (Smith et al., 2004; Harvard-Oxford cortical and subcortical structural atlases; Desikan et al., 2006). r = right; l = left; MTG = Middle Temporal Gyrus; STS/STG = Superior Temporal Sulcus/Superior Temporal Gyrus; IPL = Inferior Parietal Lobule; ITG = Inferior Temporal Gyrus; SMA = Supplementary Motor Area.

Supplementary Table 4

Overview of BOLD-responses maxima for speech-in-noise (i.e., speech task noise task condition > baseline) (p < 0.001 uncorrected for the whole brain) for information purposes only. Grey font = significant at peak level within the significant cluster (↓ indicates that the corresponding peak-coordinate is part of the cluster that is described by the cluster peak-coordinate below).

	**Speech task noise**											
	Control group						ASD group					
		x	y	z	*Z*	cluster size		x	y	z	*Z*	cluster size
STS/STG<br>TE 1.1<br>Planum Temporale	l	-39	-34	11	5.90	657	l	-45	-22	5	5.50	622
								-57	-19	8	5.26	
IFG		-57	-13	8	5.76							
		-48	-28	5	5.42							
MTG								-57	-19	-1	5.23	
							r	66	-19	5	5.70	580
								60	-13	-1	5.30	
Heschl's Gyrus<br>TE 1.0 / TE 1.1	r	45	-19	5	5.89	500		51	-16	5	5.47	
STS/STG/TE1.2		54	-4	-4	5.87							
STS/STG		57	-25	2	5.54							
Cerebellum	r	15	-76	-40	5.65	560	r	39	-64	-22	4.70	106
		18	-73	-22	5.31			27	-55	-22	4.12	
		30	-55	-28	5.22			48	-55	-28	3.54	
							r	15	-76	-37	4.50	100
								21	-61	-43	4.34	
								9	-70	-22	3.68	
	l	-27	-55	-31	4.38	88	l	-6	-70	-16	3.40	7
		33	-61	-28	4.32							
		-36	-52	-28	3.79							
	l	-15	-76	-37	4.28	65	l	-36	-67	-22	4.00	40
		-12	-76	-25	3.80			-30	-61	-28	3.80	
		-6	-79	-19	3.60							
Fusiform Gyrus								-42	-67	-13	3.39	
Dendate Nucleus	l	-12	-55	-31	4.23	11						
		-15	-49	-37	3.37							

Putamen	l	-18	2	8	5.45	235	l	-21	-1	11	3.42	8
		-24	11	8	4.51			-21	8	5	3.21	
Thalamus		-12	-16	8	4.71		l	-15	-16	2	4.12	23
	r	18	-1	14	4.41	146						
		21	11	-1	4.28							
		12	-1	5	4.15							
Insula	l	-27	20	-1	5.05	81	l	-30	26	2	4.24	41
								-30	26	-7	3.48	
							r	30	26	5	3.84	47
								42	20	-4	3.34	
IFG pars Triangularis		-36	35	2	3.26							
IFG pars Triangularis	l	-45	26	20	4.99	365						
		-48	14	29	4.93		↓	-45	23	23	4.13	
Precentral Gyrus		-42	-1	56	4.71		l	-42	2	38	4.62	183
								-42	-4	56	3.87	
							r	33	-1	50	3.43	8
pars Opercularis	r	39	17	8	4.51	45	r	51	20	29	3.51	13
Insula		30	20	-4	3.71							
	r	42	11	32	4.11	10						
Paracentral Gyrus/SFG	l	-3	14	47	4.90	191						
SMA		-3	8	59	4.36		l	-6	8	53	4.95	105
Paracingulate Gyrus		12	20	35	4.14			6	23	35	3.80	
								9	11	50	4.09	
Brainstem Inferior Colliculus	l	-6	-31	-10	4.56	78	l	-6	-31	-7	3.99	11
		-6	-22	-7	6.37							
		-6	-31	-22	5.93							
	r	9	-25	-10	4.20	21	r	9	-31	-40	3.84	14
								15	-37	-40	3.20	
	l	-3	-34	-37	4.02	18						
IPL	l	-30	-55	47	4.45	76	l	-27	-52	44	4.48	65
							r	36	-46	41	3.64	17
Angular Gyrus								39	-55	50	3.31	

Inferior Temporal Gyrus	l	-45	-55	-13	3.64	12
Fusiform Gyrus		-39	-49	-16	3.39	

Coordinates represent local activation maxima in MNI space (in mm) for the whole brain. Cluster size represents the number of voxels within a cluster. Clusters are reported that reached p = 0.001 uncorrected (peak-level) and a cluster size > 5 voxels. Regions were labelled based on a standard anatomical atlas implemented in SPM (SPM Anatomy Toolbox; Eickhoff et al., 2005). If not labelled in SPM or when the labelling was ambiguous, we additionally used atlases implemented in FSL (Smith et al., 2004; Harvard-Oxford cortical and subcortical structural atlases; Desikan et al., 2006). r = right; l = left; MTG = Middle Temporal Gyrus; STS/STG = Superior Temporal Sulcus/Superior Temporal Gyrus; SFG = Superior Frontal Gyrus; IPL = Inferior Parietal Lobule; IFG = Inferior Frontal Gyrus; SMA = Supplementary Motor Area.

Supplementary Table 5

Group comparisons of the average movement (in mm) for all directions (x, y, and z).

Average movement (mm)	ASD		Controls		*p*
	M	*SD*	*M*	*SD*	
Translation					
x	0.73	.041	0.80	0.65	0.719
y	0.80	0.68	0.68	0.50	0.590
z	1.72	0.99	1.38	0.47	0.209
Rotation					
x	0.02	0.01	0.02	0.01	0.330
y	0.01	0.01	0.01	0.01	0.956
z	0.01	0.01	0.01	0.01	0.795

Directions: x = medial-lateral axis; y = anterior-posterior axis; z = dorsal-ventral axis. Values represent the maximum movement along one axis.

Supplementary References

Alain, C., Du, Y., Bernstein, L. J., Barten, T., & Banai, K. (2018). Listening under difficult conditions: An activation likelihood estimation meta-analysis. *Human Brain Mapping, 39*(7), 2695-2709. doi:10.1002/hbm.24031

Bölte, S., & Poustka, F. (2006). *Fragebogen zur Sozialen Kommunikation (FSK)*. Bern: Verlag Hans Huber.

Bölte, S., Rühl, D., Schmötzer, G., & Poustka, F. (2003). *Diagnostisches Interview für Autismus – Revidiert (ADI-R)*. Bern: Verlag Hans Huber.

Desikan, R. S., Segonne, F., Fischl, B., Quinn, B. T., Dickerson, B. C., Blacker, D., . . . Killiany, R. J. (2006). An automated labeling system for subdividing the human cerebral cortex on MRI scans into gyral based regions of interest. *Neuroimage, 31*(3), 968-980. doi:DOI 10.1016/j.neuroimage.2006.01.021

Eickhoff, S. B., Stephan, K. E., Mohlberg, H., Grefkes, C., Fink, G. R., Amunts, K., & Zilles, K. (2005). A new SPM toolbox for combining probabilistic cytoarchitectonic maps and functional imaging data. *Neuroimage, 25*(4), 1325-1335. doi:10.1016/j.neuroimage.2004.12.034

Lord, C., Risi, S., Lambrecht, L., Cook, E. H., Jr., Leventhal, B. L., DiLavore, P. C., . . . Rutter, M. (2000). The autism diagnostic observation schedule-generic: A standard measure of social and communication deficits associated with the spectrum of autism. *Journal of Autism and Developmental Disorders, 30*(3), 205-223. doi:Doi 10.1023/A:1005592401947

Lord, C., Rutter, M., & Le Couteur, A. (1994). Autism diagnostic interview-revised: A revised version of a diagnostic interview for caregivers of individuals with possible pervasive developmental disorders. *Journal of Autism and Developmental Disorders, 24*(5), 659-685. doi:10.1007/BF02172145

Rühl, D., Bölte, S., Feineis-Matthews, S., Poustka, F. (2004). *Diagnostische Beobachtungsskala für Autistische Störungen (ADOS)*. Bern: Verlag Hans Huber.

Rutter, M., Bailey, A., Lord, C. (2003). *Social communication questionnaire (SCQ)*. Los Angeles, CA: Western Psychological Services.

Schelinski, S., Riedel, P., & von Kriegstein, K. (2014). Visual abilities are important for auditory-only speech recognition: evidence from autism spectrum disorder. *Neuropsychologia, 65*, 1-11. doi:10.1016/j.neuropsychologia.2014.09.031

Smith, S. M., Jenkinson, M., Woolrich, M. W., Beckmann, C. F., Behrens, T. E. J., Johansen-Berg, H., . . . Matthews, P. M. (2004). Advances in functional and structural MR image analysis and implementation as FSL. *Neuroimage, 23*, S208-S219. doi:10.1016/j.neuroimage.2004.07.051

von Kriegstein, K., Dogan, O., Gruter, M., Giraud, A. L., Kell, C. A., Gruter, T., . . . Kiebel, S. J. (2008). Simulation of talking faces in the human brain improves auditory speech recognition. *Proceedings of the National Academy of Sciences of the United States of America, 105*(18), 6747-6752. doi:10.1073/pnas.0710826105
